# Effects of a combined physical and psychosocial training for children with cancer: a randomized controlled trial

**DOI:** 10.1186/s12885-018-5181-0

**Published:** 2018-12-27

**Authors:** Katja I. Braam, Elisabeth M. van Dijk-Lokkart, Gertjan J. L. Kaspers, Tim Takken, Jaap Huisman, Laurien M. Buffart, Marc B. Bierings, Johannes H. M. Merks, Marry M. van den Heuvel-Eibrink, Margreet A. Veening, Eline van Dulmen-den Broeder

**Affiliations:** 1 Department of Pediatric Oncology/Hematology, VU University Medical Center, Amsterdam, the Netherlands; 2 Amsterdam Center for Innovative Health Practice, Faculty of Health, Amsterdam University of Applied Sciences, Amsterdam, the Netherlands; 3 Department of Medical Psychology, VU University Medical Center, PO Box 7057, 1007 MB Amsterdam, The Netherlands; 4 Child Development & Exercise Center, Wilhelmina Children’s Hospital, University Medical Center Utrecht, Utrecht, the Netherlands; 5 Department of Medical Psychology and Social Work, Wilhelmina Children’s Hospital, University Medical Center Utrecht, Utrecht, the Netherlands; 6 Department of Epidemiology and Biostatistics, VU University Medical Center, Amsterdam Public Health research institute, Amsterdam, the Netherlands; 7 Department of Medical Oncology, VU University Medical Center and the Cancer Center Amsterdam, Amsterdam, the Netherlands; 8 Department of Pediatric Oncology/Hematology, Wilhelmina Children’s Hospital, University Medical Center Utrecht, Utrecht, the Netherlands; 9 Department of Pediatric Oncology, Emma Children’s Hospital/Academic Medical Center, Amsterdam, the Netherlands; 10 Department of Pediatric Oncology/Hematology, Erasmus Medical Center, Sophia Children’s Hospital, Rotterdam, the Netherlands; 11 Princess Máxima Center for Pediatric Oncology, Utrecht, the Netherlands

**Keywords:** Children, Cancer, Physical exercise, Psychosocial, Intervention

## Abstract

**Background:**

Physical fitness and psychosocial function is often reduced in children during or shortly after cancer treatment. This study evaluates the effect of a combined physical exercise and psychosocial intervention on cardiorespiratory fitness, muscle strength, body composition, psychosocial function and health-related quality of life (HrQoL). In addition, intervention mediators, applicability and adherence were examined.

**Methods:**

This multicenter randomized controlled trial included 68 children with cancer [mean age 13.2 (SD: 3.1) years; 54% male] during treatment or within 12-months post-treatment. The 12-week intervention consisted of 24 individual physical exercise sessions supervised by a physiotherapist, and 6 psychosocial training sessions for children and 2 for parents. Physical fitness and psychosocial function were assessed at baseline, directly post-intervention and at 12 months’ post-baseline. Generalized estimating equations were used to simultaneously assess intervention effects at short and long-term. Additionally, we evaluated within-group differences over time. Potential physical and psychosocial mediators in the intervention effect on HrQoL were examined using the product-of-coefficient test. Applicability and adherence were assessed by trainer-report.

**Results:**

This study was able to compare 26 children who received the study intervention, with 33 children who received usual care. No significant differences in the effects of the intervention were found on physical fitness and psychosocial function at short-term. At 12-months follow-up, significantly larger improvements in lower body muscle strength (β = 56.5 Newton; 95% CI: 8.5; 104.5) were found in the intervention group when compared to the control group. Within-group changes showed significant improvements over time in HrQoL and bone density in both groups. Intervention effects on HrQoL were not significantly mediated by physical fitness and psychological function. Intervention applicability was satisfactory with an average session attendance of 67% and 22% dropout (mainly due to disease recurrence).

**Conclusions:**

This 12-week physical exercise and psychosocial training intervention for children with cancer was applicable and showed satisfactory adherence. We found no significant between-group differences in effect, except for a significant improvement in lower body muscle strength at long-term in the intervention group compared to the control group. Yet, both the intervention and the control group showed improvements in bone mineral density and HrQoL over time.

**Trial registration:**

The trial was registered at the Dutch Trial Registry (NTR1531). Registered 12 November 2008.

## Background

As a consequence of cancer and anti-cancer treatment, children with cancer have reduced physical fitness [1–3] and are at increased risk for developing cardiovascular disease [4, 5], osteoporosis [6, 7] and obesity [5], as well as depressive symptoms and anxiety [8–10]. Exercise may help to improve physical fitness and reduce the above-mentioned physical and psychological side and late effects. In the general non-cancer population, physical exercise programs have shown to increase aerobic fitness, muscle strength and bone mass in youth, reduce burden of depression in adults, and facilitate weight loss in both adults and children [11–15].

There is a large body of evidence from randomized controlled trials among adult cancer survivors that exercise can improve physical fitness and health-related quality of life (HrQoL) during and after cancer treatment [16, 17]. A dose-response relationship on these outcomes has been found for exercise intensity [18, 19]. Also psychosocial interventions have been found to improve HrQoL in adult cancer survivors [20]. However, studies evaluating the effects of exercise and/or psychosocial interventions in children with cancer are scarce. A few controlled trials with small sample sizes (*n* = 14–51) have reported that exercise can significantly improve cardiorespiratory fitness, muscle strength, flexibility and body composition in children with cancer [21–25]. Psychosocial interventions in children with cancer showed limited effects on overall level of distress for the children themselves [26–28]. However, adding a psychosocial intervention to a physical exercise program could increase the willingness and motivation to engage in physical exercise programs and may improve psychosocial functioning. To our knowledge, no studies have evaluated the effects of a combined exercise and psychosocial intervention program in children with cancer.

Therefore, this study aimed to evaluate the short- and long-term effects of the Quality of Life in Motion (QLIM) intervention (a 12-week combined physical exercise and psychosocial training intervention) primarily on physical fitness, and secondarily on psychosocial function and HrQoL, compared to a usual care control group. In addition, this study examined intervention applicability, adherence, and, by the use of mediation analyses, it aims to identify which intervention components are most important to improve HrQoL.

## Methods

### Procedure

Patients were recruited from March 2009 to July 2013 in four Dutch university hospitals: VU University Medical Center, Amsterdam; Academic Medical Center, Amsterdam; University Medical Center Utrecht, Utrecht; and Erasmus University Medical Center, Rotterdam. The Medical Ethics Committees of all hospitals approved the study. The trial was registered at the Dutch Trial Registry (NTR1531) and performed according to the 1964 Declaration of Helsinki. Eligible participants were aged 8–18 years, and were currently receiving or within the first year following cancer treatment with chemotherapy and/or radiotherapy [29]. Patients were included when the remaining treatment period included no scheduled hospitalization, and when the clinical condition (according to the treating oncologist) made it possible to exercise.

Exclusion criteria were (previous) treatment with growth hormone, due to its possible influence on bone density; stem-cell transplantation, due to the high impact of the treatment and related seriously low physical condition of the children after this treatment; cardiomyopathy, due to the impact of physical exercise on the heart condition; inability to ride a stationary bike, for testing the primary outcome; and inability to read and write Dutch, to self-reflect or to follow instructions due to learning difficulties (to be able to adequately complete the psychosocial and physical intervention). The treating physician checked the exclusion criteria by viewing medical records, school information and their own clinical impression. Both patients and their parents or legal representatives received spoken and written study information and provided written informed consent prior to participation. After baseline measurements, block randomization was performed by an independent data manager and stratified by age, gender, cancer type (haematological cancer vs. solid tumour), and treatment phase (during vs. after treatment) [29].

### Intervention

The 12-week individually performed QLIM intervention included two 45-min physical exercise sessions per week at a local physical therapy practice and one 60-min psychosocial training session once every 2 weeks for the child in the treating pediatric oncology hospital [29]. The parents also received two psychosocial training sessions; a start and evaluation session. Baseline measurements were performed in the treating hospital. The intervention started within 14-days post baseline measurement. In case of seriously low blood counts or fever, the child was instructed to postpone the physical exercise training with a maximum of one-week. As there was often need for a family break after the intense childhood cancer treatment, a one-week holiday was also accepted as a valid reason to postpone a maximum of two physical exercise training-sessions.

The physical exercise training was an individual program performed at a local physical therapy practice. This program was specifically developed for children. It included both aerobic as well as weight-bearing exercises performed in a circuit training-setting with balls, hoops, and running activities. The intensity of the physical exercise training program gradually increased.

In the first eight training sessions (the first month) the training was performed at a peak heart rate (HR_peak_) of 66–77%. During these sessions the exercise training included muscle strength training with some elements of aerobic training. In the second month (sessions 9–16) the training impact increased to a HR_peak_ of 77–90% with a training focusing more on aerobic fitness, supplemented by moderate intensive strength training. In the third month (sessions 17–24) the training included highly intensive aerobic training and strength training at a HR_peak_ of 90–100%.

All physical therapists received an instruction manual accompanied by verbal explanation. In addition to the exercise training sessions at a local physical therapy center, the intervention also included a homebased program. This home-based program started after the sixth week of the intervention. Children were instructed to perform a number of weight-bearing exercises at a high intensity-level at home at least three times per week, as advised by the physical therapist. One exercise cycle took 11-min to complete and could therefore easily be implemented as part of daily routine. The 11-min exercises were guided by timed-music to increase the joy of participation. Three to six months’ post-intervention, a booster-session was included to reinforce the importance of physical exercise, to show the children’s sport-participation to their parents and peers, and to increase self-esteem in a non-therapeutic sport setting. Children received a tennis-lesson which was preferably provided in a small group of age-matched study participants.

The psychosocial training intervention was an individualized structured program to enhance socio-emotional functioning and coping with disease-related effects. It consisted of psycho-education and cognitive-behavioral techniques including items on expression of feelings, self-perception and coping skills [30]. The intervention aimed to increase general psychosocial functioning as expressed in HrQoL, self-perception, behavior problems and depressive symptoms. The individual sessions were performed parallel to the physical exercise intervention. After each individual session home exercises on the topic of this specific session could be given to the patient if the psychologist considered it necessary. The parent sessions were scheduled at the start and end of the child’s training. The training was performed by a trained paediatric psychologist according to an instruction manual. Details of the psychosocial training, its applicability and evaluation are published elsewhere [30].

The control group received usual care according to local guidelines and preferences.

### Data collection and instruments

Measurements of all primary and secondary outcomes took place in the treating hospitals at baseline, after completion of the intervention at 4 months and at long-term (12-months) follow-up. Both the aerobic fitness and the muscle strength tests were performed by blinded assessors. The same test-equipment was used in all centres, with one exception: The Dual-energy-X-ray absorptiometry (DXA)-scanner. I.e. in all but one centres a Hologic DXA scanner with the same software was used to measure bone mineral density (BMD). In the Erasmus university hospital in Rotterdam, a Lunar scanner was used. To correct for the different scan systems, we used a transcription model to compare the data. Visits to physical therapists, sport-centres and psychologists were not structurally monitored in the control group.

### Primary outcomes

Cardiorespiratory fitness was assessed by peak oxygen uptake (VO_2peak_ expressed in ml•kg•min^− 1^) during a cardiopulmonary exercise test using the Godfrey protocol [31]. The test was performed on an electronically braked cycle ergometer with a paddling frequency of 60–80 rpm. During the test, expired air was collected, heart rate was monitored, and ventilator gas exchange data were determined breath-by-breath. The VO_2peak_ was defined as the mean score of the final 30 s of the test. Cardiorespiratory fitness data were included in the analyses for children that achieved a HR_peak_ of at least 180 beats per minute, and/or a RER_peak_ of ≥1.0.

Muscle strength was assessed using a hand-held dynamometer (CITEC; C.I.T. Technics, Groningen, the Netherlands) [32]. All children performed three repetitions (both left and right) per muscle group. The highest-score out of six repetitions was used for further analyses. Upper-body muscle strength was calculated by summing the highest-score of the shoulder, elbow and grip strength, and the sum of the highest hip, knee and ankle-dorsiflexion scores was used for lower body muscle strength.

### Secondary outcomes

Body composition was determined using percentage of fat mass (%FM) and lumbar spine (L1-L4) BMD as measured by DXA.

Physical activity was measured with an Actical accelerometer (B series, Philips Respironics Actical MiniMitter, Murrysville, PA, USA) by a 15-s time-interval and expressed as mean counts per minute [33, 34]. Mean counts per minute is a physical activity score including horizontal, vertical and depth motion scores in one end-score; higher scores indicate more activity [33, 34]. The accelerometer was attached to an elastic waist belt, and worn on the left hip during daytime at waking-hours (between 6:00 am and 11:59 pm). The accelerometer was worn on four consecutive days: Wednesday to Saturday, in the week following the measurements in the hospital. Assessing 4 days was found to be sufficient and less invasive than monitoring for one-week [35]. After wearing, participants sent the accelerometers back to the research team by postal mail. Per minute data were assessed, excluding large (> 60 min) periods of consecutive zeros to validate wear-time. A mean cpm score over the recorded days and wear-time data was derived and used in the analyses.

Fatigue was assessed with the overall-fatigue score of the child self-report version of the PedsQL™ Multidimensional fatigue scale (acute version) [36, 37]. This instrument is designed to measure both the child’s and the parent’s perception of fatigue in pediatric patients [36]. The module encompasses 3 subscales: general fatigue (6 items), sleep/rest fatigue (6 items), and cognitive fatigue (6 items), and an overall fatigue score (all 18 items). Scores were calculated according to the manual and ranged from 0 to 100 with lower scores indicating higher levels of fatigue [36]. For the present study only the overall-fatigue score was taken into the analyses. The Dutch version (8–18 year) has adequate psychometric properties and normative scores of the Dutch population are available.

Total general-HrQoL was measured with the Dutch self-report version of the PedsQL™ Generic Core Scales for children aged 8–12 and 12–18 years [36, 38]. This consists of 4 multi-item subscales: physical functioning (8 items), emotional functioning (5 items), social functioning (5 items), and school functioning (5 items). A total HrQoL score was calculated according to the PedsQL™ manual; this scale has a range of 0–100, with higher scores reflecting a better HrQoL. The PedsQL™ has proven to be reliable and valid in pediatric patients [36]. The Dutch version has adequate psychometric properties, and normative scores of the Dutch population are available [38].

Athletic competence and global self-worth were assessed with the athletic competence and global self-worth subscales of the ‘Self-Perception Profile’ for children aged 8–11 years and for adolescents aged 12–18 years [39, 40]. The Self-Perception Profile has good reliability and validity when used in children aged ≥8 years [39, 40]. Higher scores (0–100) reflect more positive self-perceptions [39, 40].

Behavioural problems were assessed in children aged ≥11 years using the Youth Self-Report with higher T-scores indicating more behavioural problems [41]. It yields a total score ranging from 0 to 100. For the present study, the total problem behaviour scale was taken into the analyses. YSR is a valid and reliable instrument to assess evaluation of internalizing and externalizing behavioural problems [41].

Depressive symptoms were assessed with the Children’s Depression Inventory [42] for children 8–18 years. The total score ranges from 0 to 100 with higher scores reflecting more depressive symptoms. Good internal consistency and test-retest reliability, and a positive correlation with clinicians’ independent global depression ratings, are reported [42].

Demographic and medical characteristics including age, gender, height, weight and body mass index, type of cancer, treatment, and treatment phase (during vs. post) were obtained from medical records.

### Adherence and applicability

Session attendance of the physical exercise and psychosocial training intervention was recorded by the therapists. The physical therapist recorded the performed training intensity (heart rate) and possible adaptations to the program on an evaluation and adaptation form. Researchers rated the applicability as ‘good’ when the program could be performed on an intensity level equal to, or < 10% lower than requested and when only small (material) adaptations were needed. The results of the home-based program were self-reported in a training-logbook for personal evaluation. As a result, no valid applicability data of this home-based intervention program were collected, and no analysis on applicability of the home-based program could be performed. Applicability of the psychosocial intervention was assessed by questionnaires which were completed by the participating psychologists and by the patients (details are published elsewhere [30]).

### Sample size calculation

Based on a previous uncontrolled study on physical exercise intervention effects on cardiorespiratory fitness levels in children with cancer (2007), the intervention group was expected to show an at least 20% greater improvement in cardiorespiratory fitness than the control group shortly after the intervention [43]. Therefore, at least 26 patients per group were required to detect an effect size of 0.8 [44] between the intervention and control group with a power of 80% and an alpha of 0.05 [29]. Taking 40% dropout into account, we aimed to include 100 patients [29].

### Statistical analysis

Data were analysed using IBM SPSS Statistics for Windows (version 20.0). Data are presented as mean (standard deviation [SD]) or median (interquartile range) for all outcomes.

Generalized estimating equations (GEE) analyses with an exchangeable correlation structure were used to simultaneously assess intervention effects on the outcome variable at short and long-term (between-group differences) [45]. This statistical method adjusts for the non-independence of observations over time. Study group, time and the interaction of study group × time were entered in the regression model as independent variables, adjusting for baseline values. The interaction term between study group and time was included to separate the short-term effects from the long-term effects. We also studied within-group changes over time using the same GEE analyses. Intervention effects were evaluated using an intention-to-treat principle. Regression coefficients with 95% confidence levels were reported for intervention effects (between-group differences) from baseline to short-term, and from baseline to long-term, and for the within-group changes over time.

In a secondary per-protocol analysis, we studied intervention effects in children who had 100% attendance to the intervention (*n* = 20) and compared them to the control group (*n* = 33).

**Fig. 1:**
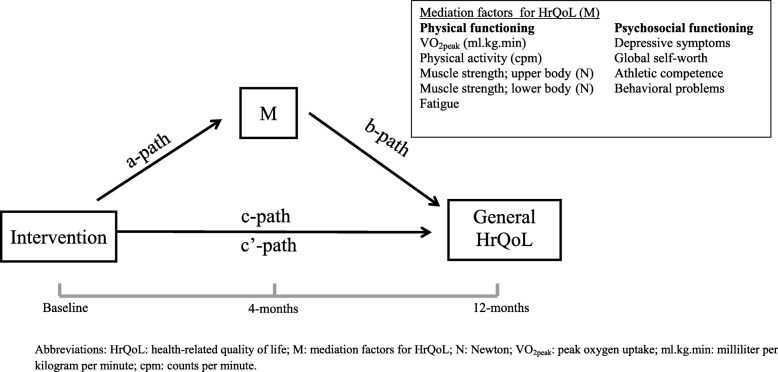
Hypothesized physical and psychosocial mediators of the intervention effect on general HrQoL. Legend: HrQoL: health-related quality of life; M: mediation factors for HrQoL; N: Newton; VO_2peak_: peak oxygen uptake; ml.kg.min: milliliter per kilogram per minute; cpm: counts per minute

To study whether the intervention effect on general HrQoL was mediated by physical fitness, physical activity, fatigue, self-perception, depressive symptoms, athletic competence, global self-worth and behavioural problems we used a series of linear regression analyses according to the products-of-coefficients test [46] (Fig. 1). First, we evaluated the intervention effect on HrQoL at the long-term adjusted for the baseline value of HRQoL (c path). Second, we evaluated the intervention effect on the potential mediator at short-term controlled for the mediator at baseline (a path). Third, the association between the potential mediator at short-term and the outcome variable HrQoL at 12 months was calculated, controlled for the intervention and baseline values of the mediator and outcome variable (b path); this step also provides information on the direct intervention effect on HrQoL at 12 months adjusted for the mediator variable (c’ path). The product of coefficients (axb) was used to estimate the relative strength of the mediation effect. We used bootstrapping techniques with 5000 bootstrap resamples to calculate the bias-corrected and accelerated 95% confidence intervals (CI) around the mediation effect (axb) using the SPSS macro provided by Preacher and Hayes [47].

## Results

**Fig. 2:**
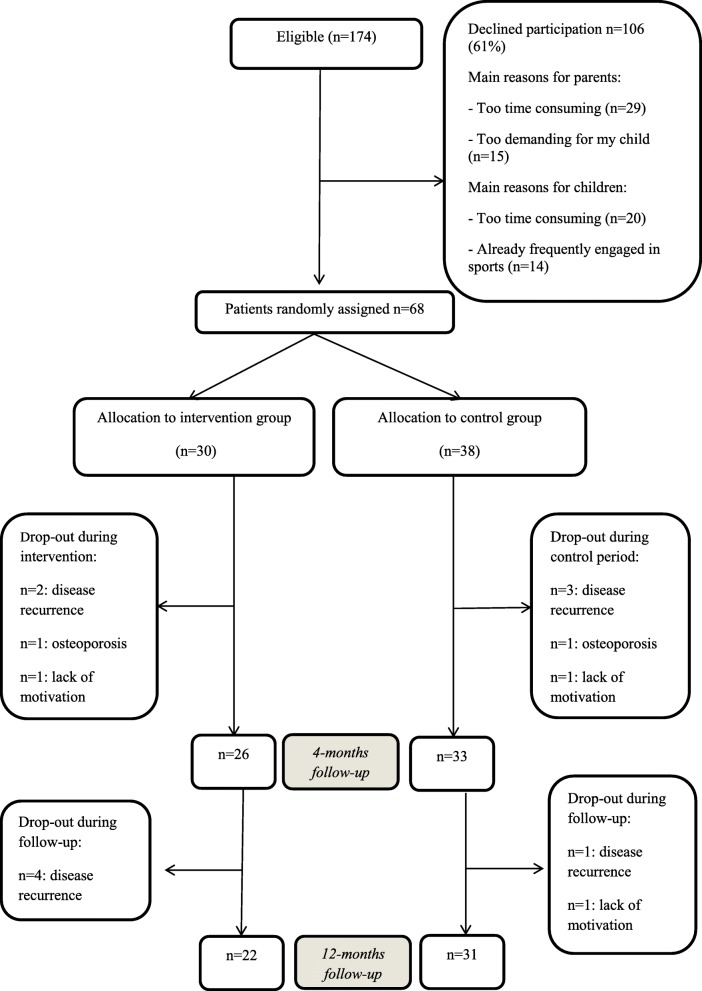
CONSORT diagram: flowchart of the study. Legend: n = number

Of the 174 eligible patients, 68 (39%) participated (Fig. 2). Mean (SD) age of the children was 13.2 (SD 3.1; range 8–18) years and 54% were male (Table 1). Thirty children were randomized to the intervention group and 38 to the control group. Half of the patients were included by the initiating centre, showing a 48% inclusion rate. Inclusion rates of the other three hospitals ranged between 31 and 39%. No significant differences were found in age, gender and medical characteristics between patients in the intervention and control group, or between participants and non-participants (Table 1) [48]. No serious adverse events were reported during the entire study. Table 2 presents the mean (SD) values for each study-outcome, per measurement.

**Table 1:** Baseline characteristics of participants in the ‘Quality of Life in Motion (QLIM) study’

	Intervention group	Control group
	*n* = 30	*n* = 38
Age, mean (SD) years	13.4 (3.1)	13.1 (3.1)
Gender, male n (%) male	16 (53%)	21 (55%)
Height, mean (SD) cm	158.9 (16.5)	154.5 (17.2)
Weight, mean (SD) kg	51.6(16.0)	49.2 (16.9)
Cancer type, n (%)		
ALL	8 (27%)	12 (32%)
AML, HL, non-HL, CML, Burkitt	12 (40%)	13 (34%)
CNS/brain tumor	1 (3%)	6 (16%)
Solid tumor	9 (30%)	7 (18%)
During treatment, n (%)	9 (30%)	12 (32%)
Lower body amputations	2 (3%)	2 (3%)
Upper body amputations	2 (3%)	1 (1%)

Abbreviations: *SD* standard deviation, *N* number, *ALL* acute lymphoblastic leukemia, *AML* acute myeloid leukemia, *HL* Hodgkin lymphoma, *non-H* non-Hodgkin lymphoma, *CML* chronic myeloid leukemia, *CNS* central nervous system

*No significant differences at baseline between the two groups

**Table 2:** Means and standard deviation scores per study outcome and measurement in the intervention and control group of the Quality of Life in Motion study

	Intervention (*n* = 30)	(*n* = 26)	(*n* = 22)	Control (*n* = 38)	(*n* = 33)	(*n* = 31)
	Pre	Post Short-term	Post Long-term	Pre	Post Short-term	Post Long-term
	mean (SD)	mean (SD)	mean (SD)	mean (SD)	mean (SD)	mean (SD)
Primary outcomes						
VO_2peak,_ (ml.kg.min)	30.1 (8.5)	31.2 (9.5)	33.8 (8.7)	31.4 (9.5)	33.0 (9.3)	35.8 (8.4)
Upper body muscle strength (N)	367.4 (114.0)	363.1 (110.2)	382.1 (95.8)	370.2 (133.7)	402.2 (148.7)	416.0 (144.5)
Lower body muscle strength (N)	587.7 (174.2)	619.8 (197.5)	660.5 (206.9)	564.0 (206.6)	595.5 (216.4)	622.0 (219.2)
Secondary outcomes						
Physical activity (cpm)	153.4 (120.1)	157.8 (81.7)	213.1 (135.3)	169.2 (97.4)	156.5 (76.8)	191.8 (110.1)
BMD lumbar spine (g/cm2)	0.78 (0.21)	0.78 (0.20)	0.83 (0.23)	0.75 (0.18)	0.76 (0.20)	0.78 (0.21)
% fat mass	31.2 (8.5)	30.1 (8.4)	31.2 (8.6)	31.0 (6.3)	29.3 (6.9)	29.2 (7.1)
General HrQoL	68.4 (18.2)	70.1 (15.7)	77.2 (16.4)	73.8 (14.1)	73.8 (17.6)	84.5 (13.1)
Fatigue	67.7 (19.8)	71.7 (17.9)	76.5 (19.9)	74.3 (15.7)	76.7 (16.9)	82.0 (17.3)
Extra outcomes used in mediation analysis						
Depressive symptoms	42.3 (31.3)	43.2 (28.7)	27.8 (29.4)	40.1 (26.2)	37.5 (27.2)	28.2 (26.7)
Athletic competence	40.1 (28.8)	40.5 (25.4)	51.9 (34.4)	39.9 (31.9)	39.8 (29.7)	38.2 (29.9)
Global self-worth	55.6 (29.6)	60.0 (28.8)	74.9 (25.9)	57.3 (24.2)	56.0 (29.3)	63.4 (32.5)
Total problems	45.7 (27.0)	48.1 (29.1)	37.5 (35.8)	41.8 (29.2)	39.3 (24.8)	32.6 (27.3)

Abbreviations: *SD* standard deviation, *n* number, *VO*_2peak_ peak oxygen uptake, *ml.kg.min*^− 1^ millilitre per kilogram per minute, *N* Newton, counts per minute, *cpm* counts per minute, *BMD* bone mineral density, *g/cm*^2^ gram per square centimetre, % percentage, *HrQoL* health-related quality of life, ^*^ *p* < 0.05, ^%^Corrected for baseline scores

### Between-group differences in primary and secondary outcomes

At short-term, no significant differences between the intervention and the control group were observed for any of the primary and secondary outcomes (Table 3). At 12 months follow-up, larger improvements in lower body muscle strength were found in the intervention group compared to the control group (β = 56.5 Newton; 95% C = 8.5; 104.5). Secondary per-protocol analyses revealed no significant differences between the intervention and the control group.

**Table 3:** Between-group differences and within-group changes over time on primary and secondary outcomes in participants of the Quality of Life in Motion study

	Between-group differences intervention group versus control group		Within group changes over time per study group	
	Short-term intervention effects ^%^	Long-term intervention effects ^%^	Intervention group ^∞^	Control group ^∞^
	β (95% CI)	β (95% CI)	β (95% CI)	β (95% CI)
Primary outcomes				
VO_2peak,_ (ml.kg.min^−1^)	−0.6 (−3.1; 2.0)	− 0.6 (− 3.6; 2.5)	2.1 (− 0.4; 4.5)	2.0 (− 0.04; 4.1)
Upper body muscle strength (N)	−20.4 (− 47.4; 6.5)	4.5 (− 21.3; 30.3)	36.8 (12.6; 60.9)*	11.8 (−8.7; 32.4)
Lower body muscle strength (N)	22.7 (− 19.8; 65.2)	56.5 (8.5; 104.5)^*^	49.1 (14.1; 84.0)*	15.3 (−24.3; 54.9)
Secondary outcomes				
Physical activity (cpm)	29.2 (−6.2; 64.8)	8.2 (−44.3; 60.7)	21.9 (−25.0; 68.9)	42.9 (8.4; 77.5)*
BMD lumbar spine (g/cm2)	−0.001 (− 0.03; 0.02)	0.007 (− 0.02; 0.03)	0.04 (0.01; 0.07)**	0.03 (0.02; 0.04)**
% fat mass	0.6 (−0.7; 2.0)	0.6 (−1.3; 2.5)	0.6 (−0.5; 1.7)	0.6 (− 0.4; 1.6)
General HrQoL	1.6 (−5.0; 8.2)	−2.5 (−9.1; 4.1)	5.6 (0.1; 11.0)*	9.7 (4.6; 14.8)**
Fatigue	−1.6 (−7.5; 4.3)	− 1.8 (− 9.8; 6.2)	3.7 (− 1.8; 9.3)	4.0 (− 1.2;9.2)
Extra outcomes used in mediation analysis				
Depressive symptoms	5.0 (−6.1; 16.1)	1.1 (− 11.3; 13.5)	−1.1 (− 2.0; 1.6)	−1.3 (− 2.4; − 0.2)*
Athletic competence	−1.3 (− 10.7; 8.1)	9.3 (− 4.4; 23.1)	10.1 (− 4.8; 25.0)	−5.2 (− 13.7; 3.4)
Global self-worth	0.04 (− 12.5; 12.6)	0.8 (− 12.7; 14.3)	7.5 (− 1.9; 16.8)	4.6 (− 3.7; 12.8)
Total problems	6.8 (− 4.8; 18.4)	3.4 (− 12.1; 18.8)	−0.4 (− 5.0; 4.1)	− 0.5 (− 4.0; 3.1)

Abbreviations: β: regression coefficient; CI: confidence interval; n of FU: number of follow-up measurements; VO_2peak_: peak oxygen uptake; ml.kg.min^− 1^: millilitre per kilogram per minute; N: Newton; counts per minute; cpm: counts per minute; BMD: bone mineral density; g/cm^2^: gram per square centimetre; %: percentage; HrQoL: health-related quality of life; ^*^ *p* < 0.05; ^%^Corrected for baseline scores; ^∞^ Changes between baseline and long-term assessment

### Within-group differences in primary and secondary outcomes

In both the intervention and control group, the bone density of the lumbar spine (intervention group: β = 0.04 g/cm^2^; 95% CI = 0.01–0.07; control group β = 0.03 g/cm^2^; 95% CI = 0.02–0.04) and general HrQoL (intervention group: β = 5.6; 95% CI = 0.1–11.0; control group β = 9.7; 95% CI = 4.6–14.8) increased significantly over time (Table 3). Additionally, in the intervention group, both the upper and lower body muscle strength significantly increased over time (upper: β = 36.8 Newton; 95% CI = 12.6–60.9; lower β = 49.1 Newton; 95% CI = 14.1; 84.0). The control group showed a significant decrease in depressive symptoms (β = − 1.3; 95% CI = − 2.4; − 0.2) and a significant increase over time in physical activity (β = 42.9 cpm; 95% CI = 8.4–77.5). However, the number of children who wore the physical activity monitor decreased dramatically over time: from 28 vs 33 at T0, to 10 vs 19 at T3 (I vs C), which makes this result less solid.

### Mediators of the intervention effect on HrQoL

We found no significant intervention effects on the potential mediators at short-term (path a) (Table 4). Fewer depressive symptoms (β = − 1.4, 95% CI = − 2.4; − 0.5), higher athletic competence (β = 0.2, 95% CI: 0.0; 0.4), higher global self-worth (β = 0.2, 95% CI: 0.0; 0.3) and less total behaviour problems (β − 0.5, 95% CI: -0.9; − 0.1) at short-term were significantly associated with higher HrQoL at long-term (b path). No significant associations with long-term HrQoL were found for physical variables and fatigue. The intervention effects on HrQoL were not significantly mediated by physical and psychosocial factors.

**Table 4:** Direct and indirect intervention effects on general Health-related quality of life, intervention effects on potential mediators, effects of the mediators on general Health-related quality of life and the univariate mediation effects

	Intervention effect on the potential mediator	Association between the potential mediator and HrQoL	Intervention effect through the mediator on HrQoL	Mediation effect	
	(a path)	(b path)	(c’ path)	(a x b path)	
	β (95% CI)	β (95% CI)	β (95% CI)	estimate (95% CI)	n
Potential mediators					
VO_2peak_ (ml•kg•min^− 1^)	−0.5 (− 3.2; 2.1)	0.3 (− 0.6; 1.1)	0.3 (− 6.5; 7.1)	− 0.3 (− 3.3; 0.8)	46
Lower body muscle strength (N)	22.7 (− 21.6;67.0)	− 0.0 (− 0.0; 0.0)	−3.3 (− 10.4; 3.8)	−0.1 (− 2.6; 0.7)	50
Upper body muscle strength (N)	−20.3 (− 48.6; 7.7)	0.0 (− 0.0; 0.1)	−2.6 (− 9.7; 4.6)	−0.5 (− 3.9; 0.5)	50
Physical activity (cpm)	29.1 (−9.8; 68.0)	−0.0 (− 0.1; 0.1)	0.6 (− 9.4; 10.7)	−0.7 (− 8.5; 1.5)	33
Fatigue	−1.2 (− 7.6; 5.1)	0.2 (− 0.1; 0.5)	−2.9 (− 10.1; 4.2)	−0.3 (− 3.7; 1.0)	49
Depressive symptoms	0.2 (− 1.6; 2.0)	−1.4 (− 2.4; − 0.5)*	−3.5 (− 9.9; 3.0)	0.7 (−2.4; 3.6)	49
Athletic competence	−1.5 (− 11.0; 8.1)	0.2 (0.0; 0.4)*	−4.2 (− 11.3; 3.0)	0.0 (− 2.8; 3.0)	46
Global self-worth	− 0.3 (− 13.2; 12.6)	0.2 (0.0; 0.3)*	−5.2 (− 12.5; 2.2)	1.0 (− 1.2; 4.8)	46
Behavior problems	1.1 (−5.1; 7.2)	−0.5 (− 0.9; − 0.1)*	0.6 (− 7.8; 8.9)	0.3 (− 6.7; 3.6)	30

Abbreviations: HrQoL: health-related quality of life; VO_2peak_: peak oxygen uptake; ml•kg•min^− 1^: milliliter per kilogram per minute; N: Newton; cpm: counts per minute; β: regression coefficient; CI: confidence interval; cpm: counts per minute; n: sample size per analyses; *p < 0.05

### Adherence

Nine (13%) participants dropped-out between baseline and short-term (4-months) follow-up, mainly due to recurrence of the disease (7/9). Six (9%) additional participants dropped-out between short- and long-term (12-months) follow-up for the same reasons (Fig. 2).

The median attendance at the physical exercise training sessions was 24 sessions (interquartile range (IQR): 20–24). Twenty out of 30 children (67%) attended all physical exercise training sessions within 12 to 16 weeks. The psychosocial training intervention was completed by 27 children (90%) [30].

A total of 23/24 children participated in the booster-session, which was planned to be a one-day group intervention. However, due to segmented patient inclusion, no more than two children could be grouped for each booster-session.

### Adaptations and applicability during the intervention

Adaptations to exercise type or intensity of the physical training intervention were reported by 54% of the physical therapists. Three material and exercise adaptations were made due to functional disabilities after a limb amputation; the seven (temporary) adaptations (shortened program, more breaks) were related to fatigue, or other disease-related symptoms of the participant. In three patient-reports, no reason for adaptation was reported. Ten children (33%) performed (some of) the exercises at a lower heart rate than described in the study manual. All others reached the requested heart rate during training. Most adaptations or intensity reductions were temporary, or related to movement restrictions after an amputation. Related to all these adaptations, we rated the applicability of the physical exercise program as satisfactory, instead of good.

According to patients and psychologists, the psychosocial training was applicable. One patient dropped-out after 20 physical exercise sessions but had, at that time, already completed the entire psychosocial intervention. In the total group, 93% of all psychosocial exercises were completed. Psychologists rated the contact with the patients, their concentration, motivation and performance of the psychosocial home exercises as good [30].

## Discussion

This randomised controlled trial describes the short- and long-term effects of a combined physical exercise and psychosocial training program on physical fitness and psychosocial function in a relatively large group of children with cancer during or shortly after treatment. In addition, potential mediators of intervention effects on HrQoL were examined, as well as adherence and applicability of the intervention. Patients from the intervention group showed larger improvements in lower body muscle strength at 12-months when compared to the control group. No other significant between-group differences were found on physical and/or psychosocial outcomes, both at short- and long-term follow-up. We found significant improvement over time in bone mineral density and general HrQoL in both groups and in both upper and lower muscle strength in the intervention group. Mediation analyses revealed that the intervention effects on HrQoL were not significantly mediated by physical and psychosocial factors. However, the significant associations between psychosocial factors and HrQoL indicate that psychosocial factors may be important intervention targets to improve HrQoL. The adherence and applicability of the intervention was satisfactory to good, for both the physical exercise and psychosocial training programs. We used one standard program for all participants to be able to compare the study groups. Our study program only allowed some adaptations. In the future, a more targeted program might be better to increase the applicability, motivation, self-worth and, at the end, the effects of the program.

We found no significant beneficial effects of the intervention at short-term. Together with the finding that both study arms improved over time, the finding suggests that adding exercise during, or shortly after the childhood cancer treatment, is unable to speed up natural recovery. However, this finding is in contrast with two other RCTs with small sample sizes that reported significant short-term intervention effects on leg and ankle muscle strength [23, 24]. In contrast to the non-significant between-group differences at short-term, we found significant larger improvements in lower body muscle strength in the intervention group at 12 months, compared to the control group. It seemed that children needed a prolonged muscle recovery period to be able to significantly increase their strength. This might be related to the extreme muscle weakness at the start of the study. Another explanation could be that the children slowly implemented their learned intervention skills into daily practice.

Some of the within-group differences over time were significant. The finding that the intervention group showed the largest increase in lower body muscle strength correlated with the between-group results on this outcome. Changes over time in physical activity were only significant in the control group. This may be related to the low number of patients who wore the physical activity monitor during the final study measurement week (ten in the intervention group and nineteen in de control group). These small numbers, combined with large individual differences in physical activity performances let to large standard deviation scores and non-significant findings, especially in the intervention group. The reason for not wearing the accelerometer was related to the discomfort of waring the monitor through a belt on the hip. Complaints especially came from girls and overweighed children.

In contrast to previous studies in adult cancer survivors [49, 50], we found that the intervention effects on general HrQoL were not significantly mediated by physical and psychosocial function. The lack of mediation effect was most likely caused by the lack of intervention effects on the mediator. The significant association found between psychosocial variables (depressive symptoms, athletic competence, global self-worth, and behavioural problems) and HrQoL indicates that those variables are important intervention targets to improve HrQoL. Future studies should aim to find more effective strategies to improve psychosocial function, as this may enhance HrQoL of these children.

To our knowledge, the present study is the first RCT with a relatively large sample size to evaluate the short and long-term effects of a combined physical and psychosocial training intervention in children with cancer. The study had a strong design and used gold-standard test methods for almost all outcomes. However, some limitations need to be considered. First, due to a lower inclusion rate than expected, the study included a total of 68 patients, instead of the planned number of 100. This implies that the study may have been underpowered to detect significant between-group differences over time. However, when performing a new power analyses using the short-term VO_2peak_ results of this study (mean VO_2peak_ 31.2 (9.5) and 33.0 (9.3) ml.kg.min^− 1^), we should have included at least 874 patients to find significant intervention effects. Therefore, it would not have been possible to find significant intervention effect even when we included the initial number of 100 subjects. Secondly, the number of patients in each study arm was skewed due to the effects of block randomisation, low patient numbers and four factor stratification rules. However, as a result of the stratification, the characteristics of both study groups were highly comparable. Thirdly, it is possible that our participants are biased towards a more positive attitude on physical and psychosocial training. Although analysis of differences between participants and non-participants showed that participants rated their physical fitness lower than the non-participants [48], we may have reached the children and parents who had the most physically active children, or were more aware of their exercise behaviours. These children might have experienced more negative effects of cancer on physical fitness and, therefore, may have rated their physical fitness lower. Children in the control group were allowed to find their own way to increase their fitness level. In the control group (apart from self-report data derived from an activity questionnaire and cost diaries) visits to physical therapists or sport centres were not monitored, possibly leading to intervention contamination. Future studies need to monitor this item more strictly, for example by asking the control group to fill out physical activity diaries throughout the intervention period, or by asking both groups to wear an activity monitor during the intervention period.

## Conclusion

Directly after the intervention this study was able to compare 26 children from the intervention group with 33 children of the control group. Results showed that performing a 12 week combined physical exercise and psychosocial training intervention is feasible for children both during and shortly after cancer treatment. However, based on these results we found no significant beneficial effects of the intervention on physical and psychosocial outcomes compared to the control group, except for improved lower-body muscle strength on the long-term. Over time, in both groups signs for natural recovery were found for bone mineral density and general HrQoL. Future research should determine whether this intervention may be beneficial to improve physical fitness and HrQoL in a much larger (European) study population with the opportunity to compare specific diagnosis or treatment-based subgroups, or when offered later in the disease trajectory. To enhance HrQoL, it may be important to improve psychosocial factors such as depressive symptoms, athletic competence, global self-worth and behavioural problems.

## Abbreviations

%FM: percentage of fat mass
ALL: acute lymphoblastic leukemia
AML: acute myeloid leukemia
BMD: bone mineral density
C: control group.
CI: confidence interval
CML: chronic myeloid leukemia
CNS: central nervous system
CPET: cardiopulmonary exercise test
cpm: counts per minute
DXA: Dual Energy X-ray absorptiometry
GEE: generalized estimating eqs.
HL: Hodgkin lymphoma
HR_peak_: peak heart rate
HrQoL: health-related quality of life
I: intervention group
M: mediation factors
N: Newton
n: number
non-HL: non-Hodgkin lymphoma
QLIM: quality of life in motion
SD: standard deviation
VO_2peak_: peak oxygen uptake
β: regression coefficient
